# Building on the 2025 National Academies Pharmacy Workshop: pharmacy deserts in the United States

**DOI:** 10.1093/haschl/qxag256

**Published:** 2026-09-22

**Authors:** Lucas A Berenbrok, Delesha Carpenter, Teresa M Salgado, Marie Chisholm-Burns, Jenny S Guadamuz, Elizabeth Hall-Lipsy, Cherokee Layson-Wolf, Whitney D Maxwell, Heidi R Olson, Megan R Undeberg, Stevie Veach, Vicki L Ellingrod

**Affiliations:** University of Pittsburgh School of Pharmacy, Department of Pharmacy and Therapeutics, Pittsburgh, PA 15261, USA; The University of North Carolina at Chapel Hill Eshelman School of Pharmacy, Division of Pharmaceutical Outcomes and Policy, Chapel Hill, NC 27599, USA; Virginia Commonwealth University School of Pharmacy, Department of Pharmacotherapy and Outcomes Science, Richmond, VA 23298, USA; Oregon Health & Science University, Portland, OR 97239, USA; University of California, Berkeley, School of Public Health, Division of Health Policy and Management, Berkeley, CA 94720, USA; The University of Arizona College of Pharmacy, Tucson, AZ 85721, USA; University of Maryland School of Pharmacy, Department of Practice, Sciences and Health Outcomes Research, Baltimore, MD 21201, USA; University of South Carolina College of Pharmacy, Department of Clinical Pharmacy and Outcomes Sciences, Columbia, SC 29208, USA; University of Illinois Chicago, Retzky College of Pharmacy at Rockford, Department of Pharmacy Practice, Rockford, IL 61107, USA; Washington State University College of Pharmacy and Pharmaceutical Sciences, Department of Pharmacotherapy, Spokane, WA 99202, USA; The University of Iowa College of Pharmacy, Department of Pharmacy Practice and Science, Iowa City, IA 52242, USA; University of Michigan College of Pharmacy, Ann Arbor, MI 48109, USA

**Keywords:** pharmacy access, healthcare access, pharmacy deserts

## Abstract

Areas with no access or limited access to community pharmacies are routinely referred to as “pharmacy deserts,” which are of growing concern in the United States. In addition to medication dispensing, community pharmacies offer a variety of healthcare services that keep populations and individuals healthy. Our objective is to present an overview of pharmacy deserts in the United States, discuss the impact of reduced pharmacy access for Americans, and propose solutions discussed at the 2025 Innovations in Pharmacy Training and Sustainable Practice to Advance Patient Care: A Workshop at the National Academies in Washington, D.C. Policy and legislation that ensure the financial sustainability of community pharmacies as staples of healthcare delivery in local neighborhoods should consider these multimodal solutions to ensure pharmacy access for all populations in the United States.

## Introduction

Areas with no access or limited access to community pharmacies are routinely referred to as “pharmacy deserts,” which are of growing concern in the United States.^1^ The ubiquity of community pharmacies was once a landscape of exemplar access to healthcare. Approximately 90% of Americans lived within 5 miles of a community pharmacy according to a 2021 analysis.^2^ However, the American public has seen recent reductions in access to local community pharmacies, including independently owned, corporate chain, and mass merchandiser locations.^3^ Nearly one-third of pharmacies in operation between 2010 and 2020 had closed by 2021.^4^

In addition to medication dispensing, community pharmacies offer a variety of healthcare services that keep populations and individuals healthy. These services include preventive care (eg, immunizations, smoking cessation, and diabetes prevention); acute care (eg, emergency contraception^5^ and treatment for minor ailments and infectious diseases); and chronic disease state management (eg, diabetes, hypertension, and dyslipidemia). However, recent changes in prescription drug reimbursement and a continued lack of insurer coverage and payment for clinical services provided by pharmacists have challenged pharmacy business models, leading to the closure of many community pharmacies including a nationwide corporate chain.^6^ Given the important contributions that pharmacies make to the US healthcare system, continued pharmacy closures could create a healthcare and medication access crisis in the United States, particularly in areas that are already underserved.

Our objective for this commentary is to present an overview of pharmacy deserts in the United States, discuss the impact of reduced pharmacy access for Americans, and propose solutions discussed at the 2025 Innovations in Pharmacy Training and Sustainable Practice to Advance Patient Care: A Workshop at the National Academies in Washington, D.C.^7^

## Defining pharmacy deserts in the United States

Pharmacy deserts characterize geographic areas where population access to community pharmacies is limited. Although many definitions of pharmacy deserts exist, most borrow elements from the Centers for Disease Control and Prevention (CDC) and the U.S. Department of Agriculture (USDA) definition of “food deserts.”^8^ Common elements across pharmacy desert definitions include US Census tracts as population areas; open-door pharmacy locations as vital points of interest; population data describing geographic areas such as urbanicity, mean annual household income, and car ownership; and driving distance thresholds from the population to the nearest pharmacy location.

The term “pharmacy desert” was first defined by Qato et al.^1^ in 2014 using both low-income and low access criteria, similar to the contemporary “low-income and low access” term used by the USDA to designate geographic areas with low access to healthy foods, formerly known as “food deserts.”^9^ In this definition, low-income communities were those wherein at least 20% of the residents had household incomes below the federal poverty level or the median household income did not exceed 80% of the median [area] household income. “Low access” denoted communities with more than 33% of the population living more than one mile from a community pharmacy (or more than half a mile for “low vehicle access” communities). Qato's initial definition of “low access” was adapted in subsequent studies to address different distance-to-pharmacy thresholds for urban (1 mile), suburban (5 miles), and rural areas (10 miles). Other pharmacy desert definitions incorporate car ownership and poverty levels of geographic regions to account for low or no access to pharmacies given transportation needs and the costs associated with travel.

Interestingly, pharmacy deserts can exist in any urban, suburban, or rural area. For example, low annual household income and low rates of car ownership in urban and suburban areas can lessen access to pharmacies given increased travel challenges (eg, time and cost) despite close proximity. Because time is a valuable yet finite resource, recent studies have incorporated drive time as an additional indicator of pharmacy access.^10^ In 2024, Sharareh et al.^10^ analyzed drive time by urban, suburban, and rural areas, given that significantly less time may be needed to drive 15 miles on high-speed roadways in comparison to 15 miles on local surface streets. However, drive-time analyses can be difficult to conduct due to a lack of accurate roadway, driving speed, and traffic data. Because of this, driving distances with thresholds by urbanicity are more commonly used than drive time in pharmacy desert definitions.

In general, rural distance-to-pharmacy thresholds allow for the longest driving distances due to increased car ownership and willingness to drive farther for necessities. Urban thresholds are the shortest for the opposing reasons. However, factors beyond urbanicity or road type should also be considered when examining pharmacy access. For example, in the largest cities in the United States, large proportions of the population, especially those with lower incomes, rely on public transportation, which can make travel to a pharmacy more difficult.^11^

## Using geographic information systems to map pharmacy deserts

Analyzing spatial relationships between 2 or more geometries (points, lines, and polygons) is the hallmark of geographic information systems (GIS) analyses. Studies mapping pharmacy deserts employ the fundamentals of GIS analysis by showing the spatial relationships between populations and community pharmacies in specified geographic areas.

To label geographic areas as pharmacy deserts, a polygon representing the boundaries of the desert shape must first be identified. Most definitions of pharmacy deserts use US Census tracts to demarcate pharmacy desert shapes.^12-15^ These shapes are then labeled as pharmacy deserts after analyzing spatial relationships, in most cases the driving distances from point to point.^16^ The starting point is a population centroid and the ending point is the nearest community pharmacy.^12^

Pharmacy deserts are well characterized in US states, metropolitan areas, and census tracts. Table 1 includes studies that have identified pharmacy deserts in the United States and presents the definitions and scope used in each analysis.

**Table 1:** Sample of pharmacy desert analyses in the United States.

Authors (publication year)	Pharmacy desert definition used	Geographic scope
Boyle et al. (2025)^12^	Low-income and low access	State (Virginia)
Chatzipanagiotou et al. (2025)^13^	Low-income and low access	State (Ohio)
Mathis et al. (2025)^14^	Travel time to the nearest pharmacy exceeds the supermarket access time for that region and urbanicity level	Country (United States)
Poondi et al. (2024)^15^	Social Vulnerability Index and distance thresholds	State (North Carolina)
Wittenauer et al. (2024)^16^	Low-income and low access	Country (United States)
Ying et al. (2022)^17^	Low-income and low access	City (New York City, New York; Los Angeles, California; Chicago, Illinois; Houston, Texas)
Wittenauer et al. (2022)^18^	Low-income and low access	State (Washington)
Guadamuz et al. (2021)^19^	Distance to the nearest pharmacy, poverty level, vehicle ownership, or combination of those factors	30 most populous US cities (census >500 000)
Qato et al. (2014)^1^	Low-income and low access	City (Chicago, Illinois)

## Causes for the rise of pharmacy deserts

Over the past decade, numerous financial challenges (eg, unpredictable reimbursements) and external factors (eg, shrinking workforce and burnout) have converged to place unprecedented financial pressure on community pharmacies.^20^ As a result, pharmacy margins on prescription drugs have substantially decreased, sometimes due to insufficient reimbursement. In worst-case scenarios, pharmacies lose money when filling prescriptions with reimbursement rates below drug acquisition costs. Such situations have made it financially unviable for pharmacies to remain open to serve the public. A recent survey of independent and grocery chain community pharmacies in rural areas found that pharmacies can experience losses of $80–$100 per prescription when filling brand medications, including commonly prescribed glucagon-like peptide-1 (GLP-1) receptor agonists.^21^

Secondly, both independent and chain pharmacies have little control over contract terms and reimbursement rates with Pharmacy Benefit Managers (PBMs), making it difficult for pharmacies to negotiate better reimbursement rates that can support and sustain a healthy business. Other strains on pharmacy business models include the use of maximum allowable costs by PBMs, which are often unpublished and unpredictable, making it difficult for pharmacies to anticipate unfair reimbursement.^22^

In addition to the lack of fair and transparent reimbursement from PBMs, pharmacists have also described “predatory insurance practices” in their communities. This occurs when insurance companies approach beneficiaries and encourage them to enroll in insurance plans with lower monthly premiums, without informing them that their local pharmacy is not part of that plan's preferred pharmacy network.^21^ When pharmacies are excluded from preferred networks, beneficiaries must switch pharmacies or pay higher costs, discouraging patients from using out-of-network pharmacies^23^ and further increasing the risk for closure of non-preferred pharmacy locations. A 2025 study showed that pharmacies that were not preferred for any Medicare Part D plan were 3.1 times more likely to close than those that were part of a preferred network.^24^

At the 2025 National Academies Pharmacy Workshop,^7^ it was suggested that independently owned pharmacies are not alone in facing the current challenges leading to the creation of pharmacy deserts. Corporately owned chain pharmacies may also struggle to maintain certain pharmacy locations due to a shrinking workforce of pharmacists and technicians working in high-paced community pharmacy environments. These workforce challenges may also contribute to pharmacy closures.

Cultural factors may also contribute to low population access to pharmacies in certain areas of the United States.^25^ Examples include limited availability of language services and culturally competent care. When pharmacies lack these characteristics, they can become functionally inaccessible to segments of communities they are meant to serve. In neighborhoods with high proportions of patients who have limited English proficiency or unique cultural health practices, pharmacies that lack bilingual staff, interpreter services, or culturally responsive counseling may not be utilized despite their close geographic proximity. This mismatch between community needs and pharmacy workforce capabilities may transform areas with nominal pharmacy presence into de facto pharmacy deserts.

These factors, in combination with limited opportunities for clinical and cognitive services (eg, comprehensive medication management) reimbursement, requisite use of mail-order pharmacies, mergers and consolidations that reduce competition, and higher operating costs are driving pharmacy closures in the United States.

## The dynamic nature of pharmacy deserts

Although patient demand for pharmacy services remains constant and may even grow as the US population ages^26^ and receives medication as a primary treatment modality, the number and location of pharmacy deserts can change over time as pharmacies close and open their businesses. When pharmacy locations close without other locations remaining open nearby, new pharmacy deserts may emerge. Conversely, when new pharmacies open in locations that do not have existing pharmacies, a pharmacy desert may disappear. These examples highlight the cross-sectional and transitory nature of most pharmacy desert studies and the need for longitudinal analyses to identify pharmacy deserts, where they are most likely to occur, and where they are likely to persist. A previous analysis identified that when pharmacy deserts increased in Chicago, they did so primarily in low-income and Black neighborhoods.^27^

Interestingly, most pharmacy desert definitions do not consider overall population growth, an aging US population requiring additional pharmacy services, or the adoption and expansion of telehealth models post-COVID-19. It is possible that growing populations could exceed the capacity of existing pharmacies, which could create other types of access issues even when pharmacies are located nearby. For example, when a pharmacy closes, individuals transition their care to nearby pharmacies that may not be equipped for a new influx of patients. Stories of longer wait times for prescription fulfillment and decreased product inventory are documented consequences from nationwide Rite Aid closures in 2025.^28,29^ In contrast, consumers may self-select other modalities for care including mail-order pharmacy or telehealth clinical visits that lessen the demand for in-person access to community pharmacies and their available services.

Lastly, it is important to note that although pharmacy closures may create pharmacy deserts, not all pharmacy deserts are created by pharmacy closures. A particular area may have sustained low access to pharmacies due to rural geography or the lack of surrounding businesses in suburban and urban areas.

## The impact of pharmacy deserts

Recent studies have shown that people living in pharmacy deserts have worse medication adherence.^30,31^ Patient proximity to pharmacies is vitally important for on-time medication pickup and access to pharmacists who can encourage medication adherence. Timely access to medications, such as antibiotics, asthma inhalers, or epinephrine pens, is also critical because delayed administration of these prescription medications may lead to negative outcomes or even death.

Medication adherence can be negatively impacted when individuals must travel longer distances to access medications, resulting in worsening management of a disease or condition. Qato et al.^30^ found adherence to cardiovascular medications, including statins, beta-blockers, and anticoagulants, decreased significantly and were sustained for at least 12 months following pharmacy closures. Similarly, a 2024 report by Anderson et al.^31^ showed a decrease in refills for anticonvulsant medications following pharmacy closures.

Community pharmacies also serve as a primary source of vaccine administration for adults. In the 2023–2024 respiratory virus season, 36.6 million influenza vaccine doses were given at community pharmacies, a 7% increase from the 2019–2020 season. In the same time period, the number of flu vaccine doses given at medical offices declined by 32%.^32^ Despite the public acceptance of pharmacies as accessible locations for vaccination, pharmacy deserts have been associated with lower receipt of influenza vaccines in persons with breast cancer^33^ and with higher vulnerability for COVID-19 infection.^18^

## Strategies proposed to address pharmacy deserts

Several strategies have been proposed to address pharmacy deserts. Such strategies aim to solve the well-documented reasons for the rising rates of pharmacy closures which, in turn, create pharmacy deserts.

### Pharmacy workforce development

Not all pharmacy deserts are a direct result of financial inviability. In some cases, pharmacies close or do not grow in numbers due to a lack of a pharmacy workforce in community pharmacy practice settings (including pharmacists, pharmacy technicians, and student pharmacists) due to burnout and well-being.^7^ Initiatives that draw new professionals and paraprofessionals to pharmacy education, training pipelines, and areas of high social vulnerability may lessen pharmacy deserts. To support our hypothesis, a previous 2021 GIS analysis showed that over half of all US community pharmacies were located in Medically Underserved Areas/Populations (MUA/MUP) or Health Professional Shortage Areas (HPSA).^34^ An additional 2021 analysis by Guadamuz et al.^19^ found that neighborhoods of predominantly Black and Hispanic/Latino populations were more likely to experience pharmacy closures than other neighborhoods.

Other initiatives that may help attract and retain individuals to the pharmacy workforce include development of pharmacy pipeline programs in vulnerable regions, tuition assistance programs, and 401 K match programs offered by pharmacy employers.^35^ Such initiatives could introduce and encourage individuals to pursue Doctor of Pharmacy degree programs and pharmacy technician training programs with reduced or no student loans.^36,37^

Federally funded programs may also attract a workforce into areas of high need, defined as areas with a low supply of professionals, high demand from communities, and/or high social vulnerability. Such federal programs may include loan repayment programs for individuals willing to work in areas of high need, including rural or underserved communities. For example, the National Health Service Corps (NHSC) offers the Rural Community Loan Repayment Program for individuals serving 3 years at an approved substance use disorder treatment facility in an HPSA.^38^ While Public Service Loan Forgiveness (PSLF) is another avenue for individuals working for qualifying public or nonprofit employers to receive loan forgiveness, it does not directly incentivize individuals to work in rural, underserved, or HPSAs. Additionally, most US pharmacies operate in for-profit models, which limits the utility of PSLF in attracting and retaining pharmacists in communities with need.

### Critical access pharmacies

Current challenges in pharmacy reimbursement and payment models make the mitigation of pharmacy deserts more complex than solely opening new pharmacies. Preventive approaches that proactively protect existing pharmacies from closing, especially in areas that rely on a single location for access, have gained interest from researchers and policymakers in the United States. These critical pharmacy locations have been defined in the literature as “keystone pharmacies” or “critical access pharmacies” (CAPs).

The “keystone pharmacy” term was first defined by Mathis et al.^14^ in 2025 as a pharmacy location whose closure would directly result in a pharmacy desert. Such a definition emphasizes the critical role a keystone pharmacy plays for a particular region and/or population and the importance of preventing a keystone pharmacy from closing. A similar yet distinct designation is the “critical access pharmacy,” borrowing from the federal “critical access hospital” designation.^39^ The “critical access pharmacy” definition has been used by states such as Oregon and Illinois to institute preferred reimbursement rates and quarterly payments, respectively.^40,41^ Advancement of federal legislation and policies that recognize CAPs and adopt initiatives that offer increased reimbursement rates to essential pharmacies would likely incentivize growth or sustain pharmacy businesses in areas of need.

Secondly, PBMs that create pharmacy networks for payers/insurers should consider including CAPs in their preferred pharmacy networks. Inclusion of CAPs in preferred pharmacy networks would incentivize patient utilization via preferred reimbursement rates. Similar approaches incentivizing pharmacy services for low-income populations exist with the Health Resources & Services Administration (HRSA) 340B Drug Pricing Program for critical access hospitals (CAHs).^42^ These models expand federal resources and allow healthcare entities to sustain business models for underserved areas in need of critical resources such as prescription medications.

### Provider status and reimbursement

Historical pharmacy business models have been primarily driven by revenue associated with dispensing prescription medications. More recently, community pharmacies have sought to diversify revenue by charging for clinical services. While pharmacies have continued to innovate and expand clinical service offerings within state-specific scopes of pharmacy practice, payers have been slow to recognize pharmacists as providers who can bill and receive reimbursement for services rendered. However, recent movements on both state and federal levels have been positive.

As of June 2024, 36 states have enacted legislation to allow pharmacists to bill for services with state Medicaid or commercial payers.^43^ And for over a decade, professional pharmacy organizations have advocated for pharmacist provider status to receive federal recognition of pharmacists’ ability to bill for services. Advancements to such advocacy were accelerated with the COVID-19 pandemic, which resulted in pharmacists’ ability to administer COVID-19 vaccines, test for COVID-19, and initiate treatment for COVID-19 in addition to many other legislative priorities and changes allowing advanced roles for pharmacy technicians and student pharmacists.

### Pharmacy workflow transformation

When coupled with provider status and additional opportunities for reimbursement, innovation and automation in the prescription dispensing process may serve as a potential solution to mitigate pharmacy closures. For example, automated dispensing at central fill locations has been implemented by national and regional pharmacy organizations to outsource routine tasks from brick-and-mortar stores in exchange for pharmacy team participation in other revenue-generating clinical services. Transferring these routine tasks, such as inventory management, prescription preparation, and billing, to more efficient off-site locations has the potential to transform the business models of brick-and-mortar pharmacies toward sustainable, higher-value clinical service offerings.

### Mail-order pharmacies

It is also important to note that pharmacy deserts cannot be solved with single-modal solutions. An example of a single-modal solution is for mail-order pharmacies to serve as a comprehensive replacement of closed community pharmacies. While mail-order pharmacies may be able to reach certain geographic areas of the United States by mailing prescription medications, filling the gaps of community pharmacy closures also necessitates a solution to replace the in-person patient care services that closed community pharmacies formerly offered, such as vaccinations.

Vaccination services require trained healthcare professionals such as a pharmacist or student pharmacist, and in many states, a pharmacy technician.^44^ Other examples of patient care services community pharmacists offer that require in-person visits for assessment include blood pressure monitoring, contraception care, test and treat services, and administration of long-acting injectable medications (eg, long-acting antipsychotics, medroxyprogesterone). Therefore, community access to pharmacy professionals practicing in community pharmacies remains paramount.

Other services not replicated by traditional mail-order pharmacies but available at brick-and-mortar pharmacies include antibiotic and controlled substances dispensing; self-care consultations and treatment with over-the-counter medications and dietary and herbal supplements, non-prescription medical devices and equipment, and diagnostic tests for home use; direct assessment of patient administration technique (eg, inhalers and injections); and delivery of chronic disease state management and education (eg, cardiovascular disease and diabetes). Mailed medications may not be preferred by patients due to privacy/security concerns and delays due to recent US postal restructuring and consolidation.^45^ Patients risk theft of their medications that are delivered to publicly accessible mailboxes or are delivered without a required signature.^46^ Lastly, maintaining and regulating temperatures of mailed medications may pose further challenges.

### Direct-to-patient programs

Another proposed solution to expand patient access to prescription medications includes direct-to-patient (DTP) programs, which have emerged from pharmaceutical manufacturers. Example programs include GLP-1 agonists, immunotherapy products, anticoagulants, insulin, and asthma inhalers. However, DTP programs alone cannot remediate pharmacy deserts as a single-modal solution.

First, no healthcare professional can comprehensively view and assess a patient's complete medication regimen idependently when multiple sources of medication procurement (eg, multiple pharmacies and DTP manufacturer models) are used. This fractionation of care compromises patient safety by increasing risk of medication errors, drug–drug interactions, and misprescribing.

Second, no manufacturer program can be a comprehensive source for all of a patient's medication needs. Most DTP programs include expensive brand medications, leaving patients to utilize other traditional sources for their generic prescription medications. Although cost savings to patients may increase the likelihood of better patient adherence, patient safety may remain at risk if a comprehensive medication list is not available to the patient's entire healthcare team.

Lastly, our belief is that DTP programs operate similarly to online or mail-order pharmacies with the same limitations to the comprehensive care provided (eg, limits in dispensing certain medication classes or providing clinical services), further supporting the need for multimodal solutions to protect and ensure public access to pharmacies and pharmacy services.

## Conclusions

Pharmacy deserts are well characterized in the United States, and as they continue to grow, patient access to medications and to community pharmacy-based healthcare services will decrease, negatively impacting patient health outcomes. Policy and legislation that ensure the financial sustainability of community pharmacies as staples of healthcare delivery in local neighborhoods should consider multimodal solutions to ensure pharmacy access for all populations in the United States.

## Supplementary Material

qxag256_Supplementary_Data
